# Gastrulation and Body Axes Formation: A Molecular Concept and Its Clinical Correlates

**DOI:** 10.21315/mjms2022.29.6.2

**Published:** 2022-12-22

**Authors:** Razif Abas, Siti Saleha Masrudin, Ahmad Mukifza Harun, Noorkardiffa Syawalina Omar

**Affiliations:** 1 Department of Human Anatomy, Faculty of Medicine and Health Sciences, Universiti Putra Malaysia, Selangor, Malaysia; 2 Department of Anatomy and Embryology, Faculty of Medicine, Leiden University Medical Centre, Leiden, The Netherlands; 3 Faculty of Engineering, Universiti Malaysia Sabah, Sabah, Malaysia; 4 Department of Obstetrics and Gynaecology, Faculty of Medicine, Universiti Teknologi MARA, Sungai Buloh Campus, Selangor, Malaysia; 5 Department of Obstetrics and Gynaecology, Faculty of Medicine, Universiti Kebangsaan Malaysia, Kuala Lumpur, Malaysia

**Keywords:** embryology, gastrulation, body axis, molecular regulation, anomaly

## Abstract

During the third week of human pregnancy, an embryo transforms from two germinal disc layers of hypoblast and epiblast to three germinal layers of endoderm, mesoderm and ectoderm. Gastrulation is a complex process that includes cellular mobility, morphogenesis and cell signalling, as well as chemical morphogenic gradients, transcription factors and differential gene expression. During gastrulation, many signalling channels coordinate individual cell actions in precise time and location. These channels control cell proliferation, shape, fate and migration to the correct sites. Subsequently, the anteroposterior (AP), dorsoventral (DV) and left-right (LR) body axes are formed before and during gastrulation via these signalling regulation signals. Hence, the anomalies in gastrulation caused by insults to certain molecular pathways manifest as a wide range of body axes-related disorders. This article outlines the formation of body axes during gastrulation and the anomalies as well as the clinical implications.

## Introduction

The term ‘gastrulation’ means the formation of gastrula, a hollow cup-shaped structure. In embryology, gastrulation is a process in which an embryo transforms from a bilaminar germinal disc (hypoblast and epiblast) to a trilaminar germinal disc (endoderm, mesoderm and ectoderm) during early development. This transformation takes place during the third week of human gestation. Gastrulation subsequently enables the establishment of germ cell layers, which marks the commencement of systematic organogenesis. Gastrulation is also a method for developing a multi-layered body plan that demarcates anatomical axes (1).

Gastrulation is a complicated process involving cellular mobility, morphogenesis and cell signalling including gradients of chemical morphogens, transcription factors and gene expression differences. Human peri-implantation development has been poorly understood due to legal and ethical constraints on the study of human embryos, as well as a paucity of materials. In contrast to mouse gastrulation, human gastrulation research is mostly descriptive or based on assumptions rather than concrete facts (2).

The aim of this review article is to elaborate the process of gastrulation with the formation body axes, as well as its anomalies in terms of molecular regulation and its clinical correlates.

### Gastrulation Event

The emergence of the primitive streak, a channel in the epiblast layer’s caudal end, marks the start of gastrulation (3). The cranial-caudal axis is thus firmly established by the creation of a primitive streak. A swelling of cells along the connecting stalk causes the primitive streak to form. The swelling lengthens and takes on a linear shape. Subsequently, the embryo’s cells proliferate and move toward the midline. The embryo’s cranial end appears to play a significant role in the start of the gastrulation process. The cells in the epiblast approach at a faster rate near the primitive streak’s cranial end, generating the primitive pit, a circular depression. As the primitive streak and pit lengthen, migratory epiblast cells join the primitive streak and pit at the cranial end, generating the primitive node. Epithelial cells on the epiblast layer’s lateral border undergo a cellular transformation from epithelial to mesenchymal, enabling them to separate and move into the primary streak (4).

The cells that travel along the primitive streak merge into the innermost layer and develop into endoderm, one of the three derivative germinal layers. The uppermost group of cells to separate and invaginate will insert into the gap between the epiblast and endoderm to develop into the mesoderm, which is the second layer. Lastly, epiblast cells will develop into ectoderm, the outermost layer (5). As the embryo develops, cell proliferation and invagination occur in both directions; nevertheless, from the caudal end, the primordial streak continuously extends in one direction, then reverse the direction. After the complete development of the intraembryonic mesoderm, regression of the cell proliferation occurs and by the end of the fourth week, the primitive streak should have vanished completely (5). Interestingly, the epiblast’s migration and invagination along the primitive streak have been mapped and their eventual fates, such as axial mesoderm, paraxial mesoderm, intermediate mesoderm and lateral plate mesoderm, have been determined (5).

The freshly developed trilaminar germ disc is ready for the creation of organ systems after the formation of the three distinct germinal layers. The event which direct interaction and induction between these three layers are essential for the disc to function. Cells continue to invade through the primitive pit, which is now known as the primitive node. The notochordal process is formed when cells create a furrow tube stretching from the cephalic end to the prechordal plate. The notochordal process develops longer as the embryo grows in either direction until it merges with the endoderm to produce the notochordal plate.

The neurenteric canal is a free conduit between the gestational sac and the amniotic space once the fusion is complete (6). The neurenteric canal is thought to originate to maintain pressure balance between the two chambers. The two corners of the notochordal plate will join later in development, forming a notochord that consist of a solid rod mesoderm. Notochord is a structure in the mesoderm that serves as both structural support and a marker for the embryo’s midline (1).

### Molecular Regulation and Clinical Correlates

Gastrulation in vertebrates is characterised by enormous cell movements that create and form the germ layers. Various signalling channels tightly coordinate individual cell actions in time and space during gastrulation. These channels direct the proliferation, shape, destiny and migration of cells to their right locations. During gastrulation in the vertebrates, the body of the embryo is shaped primarily by convergence and extension movements (7). Before and during gastrulation, the anteroposterior (AP), dorsoventral (DV) and left-right (LR) body axes are created. The establishment of these body axes is signalled by a molecular signalling regulation. Anomalies in the gastrulation process are caused by aberrations of certain molecular pathways. The aberrations will manifest in a range of disorders with clinical correlates.

### Anteroposterior Body Axis

Continuous production and distinct organisation of axial and paraxial mesoderm are required for AP body axis elongation. Cells arising from the anterior most of the primitive streak create the axial mesoderm, which later develops into the notochord, according to fate-mapping studies (8). Head creation in the mouse embryos is dependent on signals produced by two organising centres during gastrulation, the anterior visceral endoderm (AVE) and early gastrula organise (EGO). EGO is also called anterior primitive streak. The molecular regulation of the signals that activate anterior neural development from the epiblast, on the other hand, is not well understood.

Aside from their role in nutrient intake and delivery, visceral endoderm cells are involved in the cavitation process, which causes the central amniotic cavity to form in the embryonic ectoderm. AVE plays a role in preventing signals that promote posterior development from reaching the anterior embryonic areas. In addition, a genetic cascade involving *hepatocyte nuclear factor 3β* (*HNF3β*), *mothers against decapentaplegic homolog 2* (*SMAD2*), *LIM1*, *orthodenticle homeobox 2* (*OTX2*) and *activin receptor type-1B (ActR1B)* in the AVE results in the generation of secreted transforming growth factor-β (TGFβ) antagonists, *Cerberus 1* (*Cer-1*) and *Letty1*, that prevent *Nodal* signalling from reaching the epiblast in the anterior region (9). It has been shown that the transcription factors *LIM1* and *OTX2*, as well as the TGFβ superfamily protein *Nodal*, play crucial functions in the proper prospective forebrain area of the development in the visceral endoderm.

### Dorsoventral Body Axis

**Figure 1:**
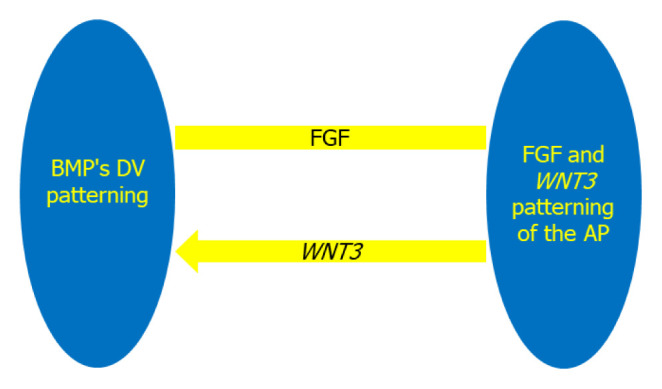
The establishment of the DV and AP axes is controlled by interconnected regulatory mechanisms. The DV axis is determined by BMP signalling, which induces ventral fate, whereas the FGF and *WNT3* signalling pathways control AP patterning. *WNT3* stimulation, on the other hand, also promotes BMP signalling

Bone morphogenetic protein 4 (BMP4), a subgroup of the TGFβ family, is produced all across the embryonic disc. BMP signalling is assumed to affect *Nodal* and *WNT3* expression and it has been proposed in vitro (10). On the ventral side, BMP and *WNT3* ligands enhance the ectoderm’s epidermal fate. The DV axis is determined by BMP signalling, which induces ventral destiny, whereas the fibroblast growth factor (FGF) and *WNT3* signalling pathways also control AP patterning, as interconnecting (Figure 1). Hence, the abnormalities of *Zbtb14* mutant mice could be owing to a misalignment of BMP and *WNT3* signalling as shown in the previous study (11). FGF binds to and activates FGF receptors (FGFR) in addition to BMP4 which later promotes DV and AP formation via *FoxB1* inhibition. FGF binding causes receptor dimerisation and intracellular trans phosphorylation in FGFR, which is a receptor tyrosine kinase (RTK). This FGF expression is restricted to the dorsal border and aids in the development of the DV axis by stimulating the dorsal organiser. The dorsal cell determinants are protected by the organiser by activating *noggin*, *follistatin* and *chordin*, in the dorsal area and suppressing the process of transcriptional repressors of dorsal genes *vox/vent/ved* (12). Additionally, these proteins will later cause the mesoderm to ventralise, resulting in the formation of kidney, blood and body wall mesoderm.

Other signalling molecules, in addition to *Nodal*, have a role in posterior epiblast growth. The *WNT* signalling pathway, in particular, has been implicated in the development of primitive streaks. *WNT3* homozygous mutant embryos lack mesoderm tissues and do not generate a primitive streak. Furthermore, promoter analysis revealed that the *WNT/β-catenin* pathway targets the *Brachyury (T)* gene, which is expressed in notochord precursor cells. *Brachyury* serves as a ventral and dorsal mesoderm marker. *Brachyury*, which indicates the uncommitted mesoderm and notochord at this stage of gastrulation, along with *goosecoid*, which indicates the prechordal plate, allow the main territories of the gastrula to be identified (13). This *Brachyury* gene also is required for cellular expression through the primitive furrow and affects the formation of mesodermic dorsum in the central and tail regions of the embryo. *Brachyury* is expressed in the tailbud at later stages and, when combined with *Xnot-2*, allows for the study of the growing tail. As a result, in addition to *Nodal* inhibitors, the AVE could also be a source of anti-*WNT* signalling molecules. Thus, it is plausible that AVE-derived signals act on epiblast tissue to counteract *WNT* signalling, which is implicated in the creation of primitive streaks (9).

### Left-Right Body Axis

While extrinsic clues such as weight inertia or sperm entrance point can set the AP and DV axes, there is no autonomous mechanism to determine the right or left direction because no known gross anatomy component of description distinguishes right from left or vice versa (14). Along the whole dimension of the notochord, runs by an electric stream, which may have oriented LR asymmetry during embryonic development, according to a prior study (15). As a result, a magnetic vector pointing either to the L or R can be measured on the dorsal or ventral sides of the embryo.

Node and primitive streak secrete FGF8, which causes *Nodal*, a subgroup of the TGFβ family, to be disclosed on the left side near the node. Subsequently, *Nodal* and *Lefty-2* are expressed in the extreme sides of the mesoderm, while *Lefty-1* is designated on the left side of the neural tube’s ventral area, as the neural plate is induced (16). Induction of these three genes is also aided by the *Brachyury (T)* gene development, which is produced in the area surrounding the notochord. The *Lefty-2* and *Nodal* expressions, in turn, positively feedback the transcription factor *PITX 2* expression, hence, establishes left sidedness via downstream effectors. *Sonic hedgehog (SHH)*, which is also found surrounding the notochord, may act as a central boundary, obstructing the overexpression left-sided genes at the right-sided area (17). Furthermore, *Snail* expression may also influence downstream associates genetically in the establishment of right sidedness.

### Caudal Dysgenesis

Caudal dysgenesis (CD) is a congenital disorder in which the foetal development of the lower spine—the caudal partition of the spine—is abnormal. It occurs at a rate of approximately one per 60,000 live births. It has been suggested that CD is caused by anomalies during the development at the posterior part of the mesoderm. An embryological disturbance occurs prior to the fourth week of intra uterine life when the structures at the caudal part of the embryo are growing. The nature of the disturbance is unknown, even though maternal diabetes, hereditary factors and hypoperfusion have been proposed as possible reasons. CD affects as much as 1% of diabetic mothers’ pregnancies. In some CD patients, an aberrant abdominal artery has also been reported. The majority of CD cases are sporadic, similar to sirenomelia, with the possibility that each occurrence is an autosomal dominant disorder generated by a new occurrence of unplanned mutation (18).

A prior study found that normal posterior development requires the simultaneous reaction of *CDX* genes, sustained WNT signalling and *HOX* genes activation at the right time (19). By altering the posterior growth zone, a change in any of these characteristics induces axial growth arrest. Caudal development requires proper retinoic acid (RA) signalling. Excessive RA injection to pregnant mice causes caudal deformities comparable to human CD, as well as sirenomelia in the majority of the survivors. Axial truncations are also caused by the loss of the RA-degrading enzyme *Cyp26a1* gene, which acts on a similar route. Haploinsufficiency of NOTCH signalling in combination with short-term foetal hypoxia, which disrupts FGF signalling, greatly enhances the penetrance and severity of vertebral abnormalities (20). Hence, the phenotypic penetrance of genetically sensitive embryos in CD could be affected by a comparable environmental insult.

### Sirenomelia

Sirenomelia is a relatively unusual condition in which the foetus resembles a mermaid due to the fusion of the lower extremities. More severe problems, such as gastrointestinal and urogenital abnormalities, are frequently linked to the condition. The reported prevalence in the population varies from 1 per 60,000 until 1 per 100,000, with a female to male bias of 1:2.7 (18). Limb fusions come in a variety of severity. Seven kinds of the sirenomelia’s classification have been established based on the development of the ossification or osteogenesis (I: all thigh and leg bones present, II: single fibula, III: absent fibulae, IV: partially fused femurs and fused fibulae, V: partially fused femurs and absent fibulae, VI: single femur and tibia, and VII: single femur and absent tibiae) (21). Furthermore, instead of the usual two umbilical arteries, affected foetuses frequently only have one. The origin of this artery, which is derived from the vitelline artery, is abnormally high in the abdominal cavity. Surprisingly, sirenomelia is rarely linked to diabetes in the mother.

The BMP pathway is another signalling system linked to sirenomelia in mice. Sirenomelia is always present in *Bmp7/Twsg* double mutants. Twsg (twisted gastrulation) is a BMP regulator that regulates BMP signalling by either promoting or inhibiting it. While single insult is unharmed, multiple insults are embryonically fatal and show sirenomelia, demonstrating that while deficiency of *Bmp7* may be overcome by other BMP ligands, the deficit of more than one copy of *Twsg* reduces BMP signalling sub-threshold which is required for normal morphogenesis. Furthermore, increased expression of *noggin* in the chicken embryos’ caudal end frequently results in a fused limb phenotype, implying that BMP signalling is required for tail ventral mesoderm development. Furthermore, the posterior FGF8-expressing area and the tailbud mesoderm expressing *T* are reduced in *Bmp7/Twsg* compound mutants. A reduction in the amount of invaginating cells towards the ventral ectodermal ridge by the event of low BMP levels was also postulated as a feasible cause of the serious phenotype (18).

### Currarino Syndrome

Currarino syndrome (CS) is a trio of caudal abnormalities that include sacral bone abnormalities, presacral tumour and anorectal malformation; CS is essentially caudal split cord malformations. Toward the end of gastrulation, the unsuccessful separation at the DV caudal eminence from the hindgut endoderm is thought to be the cause of Currarino’s triad abnormalities. The type of presacral mass can range from cystic lesions like a dermoid cyst and anterior meningocele, to solid tumours like mature teratomas or benign hamartomas. CS is inherited in an autosomal dominant form, while spontaneous occurrences are common. In 90% of familial instances, but only half of the sporadic cases, a mutation in the *MNX1* gene has been discovered. A presentation of CS alongside split cord malformation has been documented (22). The documentation was proof that CS is a frequent embryogenic genesis due to unsuccessful caudal eminence DV separation. The separation occurs at the completion of the gastrulation stage, from the hindgut endoderm.

Only the CS, a congenital caudal deformity in humans, has been associated with a mutation in the gene of *MNX1* homeobox (previously *HLXB9*). The presence of ectopic *MNX1* expression in mice RECC/19 embryos could explain the presacral neural-derived bulk. This presacral mass is a defining feature of CS and it is worth observing that *HLXB9* mutations are linked to CS/autosomal-dominant sacral agenesis, implying a connection (23). Neural cell adhesion molecule (N-CAM) and liver cell adhesion molecule (L-CAM), as cellular adhesion molecules, are associated with the separation and aggregation of multiple types of tissue and are typically elevated during gastrulation; it is postulated that a genetic insult in the expression of CAM causes aberrant disengagement of the caudal bud from the endoderm (22).

### Conjoined Twinning

Rarely, embryo duplication occurs later in the gastrulation period, with the creation of two distinct primitive furrows. The uppermost bilaminar layer and amniotic cavity have already been created at this period, hence the inadequate separation forming monochorionic and monoamniotic twinning. Any subsequent attempts to detach the twins will lead to imperfect separation, resulting in conjoined twins (24). Relatively, 30% of conjoined twins are united at the rump or pelvic outlet as pyopagus or ischiopagus, respectively, with concurrent spinal cord fusions. In up to 75% of instances, there are fusion or form anomalies in the vertebrae (25).

Human LR patterning problems are linked to the zinc finger transcription factor (*Zic3)* mutations and mice lacking *Zic3* have a similar phenotype. A *Zic3* deficiency causes axis duplication in varying degrees, which manifest in several forms, from the development of a partial secondary notochord to conjoined twinning, the most extreme form (26). Furthermore, the *Pitx2/cVg1(growth differentiation factor 1)/Nodal* pathway could be a contender for explaining armadillos’ obligatory quadruplets or the increased prevalence of conjoined and monozygotic twins in some populations (27). As its laterality, conjoined twins are produced when a secondary left-sided axis is created by the accessory expression of *siamois* or *β-catenin*, and they follow the general pattern of ‘left twin normal, right twin random’ as shown in twins induced by *Xwnt8*. Secondary axis produced by *siamois* or *β-catenin* is linked to a secondary LR coordinator, which then suppresses any signals coming from the left twin (28).

### Situs Inversus and Kartagener Syndrome

Situs inversus is a laterality condition in which the internal organs are inversely located in the abdominal and thoracic areas. The organs do not have their characteristic LR asymmetry pattern. The visceral organisation in situs inversus is a mirror reflection of normal anatomy. A pulmonologist previously documented a small number of cases with the trio of situs inversus, sinusitis and bronchiectasis (29). A relationship between ciliary abnormalities and situs inversus was discovered when a researcher discovered cilia immotility in infertile men, half of whom had Kartagener’s trio (29). Cilia were thought to play a part in determining laterality. Situs inversus is usually derived from signalling deficiency at the primitive node engaging function and structure of the cilia. Kartagener syndrome is a subgroup of primary ciliary dyskinesia (PCD) and represents about 20% of ciliopathies. However, situs inversus linked with PCD is almost unrelated to autosomal dominant disease (30). PCD patients typically have issues with expelled mucociliary, resulting in sinusitis, bronchiectasis, or chronic otitis media.

PCD is most typically caused by mutations in *DNAH5*, however, mutations in *DNAI1* and *DNAH11* can also cause it. All are components of the *dynein* outermost arm, which are required for ciliary action surrounding the node. In about half of the patients, mutations in these genes cause randomised situs, which leads to situs inversus (30). During gastrulation, a *Nodal*, a member of the TGFβ family, is necessary for the development of the primitive streak. A structure assumed to be the useful counterpart of the chick and frog ‘organiser’ and in mesodermal cells of lateral origin, an allele of *Nodal-lacZ* reporter displayed *Nodal* expression asymmetrically. Furthermore, *HNF3-β^+/−^ Nodal^1acz/+^* embryos of double-heterozygous with *Lacz* property on both sides manifest frequent abnormalities in body situs (31). In the mouse mutants, which demonstrated a situs inversus, the inversion sites of *lefty* expression indicate a downstream of *lefty*.

### Sacrococcygeal Teratoma

In most cases, the primitive streak vanishes completely. Occasionally, traces of the primitive streak can be found in the sacrococcygeal regions. These arrays of the pluripotent cells multiply hence produce tumours known as sacrococcygeal teratoma (SCT). SCT are the most frequent extragonadal germ cell tumours (EGCCT) in newborns, occurring in about 1 in every 27,000 live births, with a 3–4:1 female to male ratio, which can turn malignant (32). These tumours frequently contain tissue derivatives of all three germ layers because the primitive streak contains pluripotent cells. SCT is usually detected during infancy or at birth. Alpha-fetoprotein (AFP) is produced by the yolk sac, foetal liver and foetal gastrointestinal tract, hence serum AFP levels are usually increased at this time (32).

Several oncoproteins and tumour suppressor proteins, such as ras, fos, and jun, nm23 and p53, are expressed by SCT. However, no link was seen between the strength of their expression and tumour size, age or patient survival rate. There is also no association between the mature and immature types of tumours (33). In 11 prepubertal and five post-pubertal adult teratomas, as well as immature prepubertal sacrococcygeal teratomas, chromosome 12p changes, including i(12p), were found to be absent. The absence suggests that, when compared to post-pubertal gonadal counterparts, i(12p) may be less relevant in the aetiology of EGGCT, highlighting higher proximity to prepubertal gonadal neoplasms (34).

## Conclusion

**Figure 2:**
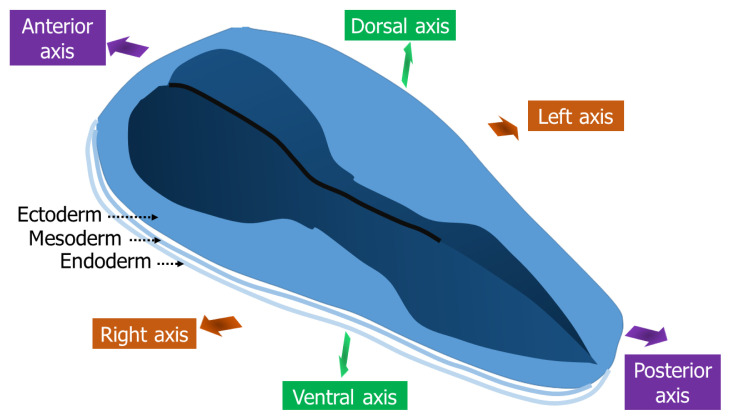
The directions of three-body axes at the end of gastrulation event: AP, LR and DV. Three germinal layers are also developed upon completion of the gastrulation process; ectoderm, mesoderm and endoderm

The gastrulation event which occurs during the third week of embryological development plays an important role in producing three germinal layers: the ectoderm, mesoderm and endoderm. These germinal layers later determine the future adult derivatives (organogenesis). Furthermore, the body axes establishment such as AP, DV and LR are also orientated during this event (Figure 2). Each establishment is controlled by a different collection of molecules and their molecular pathways, and each pathway results in different cell behaviour that helps shape the embryo properly (Table 1). The insults of specific molecular pathways hence cause an anomaly in gastrulation events. Subsequent clinical correlates with the anomalies are manifested by a variety of diseases.

## Figures and Tables

**Table 1:** Basic molecular regulation in body axis formation during gastrulation

	AP body axis	DV body axis	LR body axis
Molecular involvements	*HNF3β*	BMP4	FGF8
	*SMAD2*	*Nodal*	*Nodal*
	*LIM1*	*WNT3*	TGFβ
	*OTX2*	FGF	*Lefty-2*
	ctR1B	*Noggin*	*Lefty-1*
	Cer-1	*Follistatin*	*Brachyury*
	Letty1	C*hordin*	*PITX 2*
	*LIM1*	*Vox/vent/ved*	*Sonic hedgehog*
	*OTX2*	*Brachyury*	
	TGFβ	*Xnot-2*	
	*Nodal*		
